# Biological Self-Healing of Cement Paste and Mortar by Non-Ureolytic Bacteria Encapsulated in Alginate Hydrogel Capsules

**DOI:** 10.3390/ma13173711

**Published:** 2020-08-22

**Authors:** Mohammad Fahimizadeh, Ayesha Diane Abeyratne, Lee Sui Mae, R. K. Raman Singh, Pooria Pasbakhsh

**Affiliations:** 1 School of Engineering, Monash University Malaysia, Bandar Sunway 47500, Malaysia; Mohammad.Fahimizadeh@monash.edu (M.F.); ayeshaabeyratne@gmail.com (A.D.A.); 2 School of Science, Monash University Malaysia, Bandar Sunway 47500, Malaysia; lee.sui.mae@monash.edu; 3 Department of Chemical Engineering, Department of Mechanical and Aerospace Engineering, Monash University, Clayton 3168, Australia; raman.singh@monash.edu

**Keywords:** reinforcement, CaCO_3_, physical properties, concrete, biomineralization, self-healing, cement composite, durability, microbially induced calcium carbonate precipitation, super absorbent polymer

## Abstract

Crack formation in concrete is one of the main reasons for concrete degradation. Calcium alginate capsules containing biological self-healing agents for cementitious materials were studied for the self-healing of cement paste and mortars through in vitro characterizations such as healing agent survivability and retention, material stability, and biomineralization, followed by in situ self-healing observation in pre-cracked cement paste and mortar specimens. Our results showed that bacterial spores fully survived the encapsulation process and would not leach out during cement mixing. Encapsulated bacteria precipitated CaCO_3_ when exposed to water, oxygen, and calcium under alkaline conditions by releasing CO_3_^2−^ ions into the cement environment. Capsule rupture is not required for the initiation of the healing process, but exposure to the right conditions are. After 56 days of wet–dry cycles, the capsules resulted in flexural strength regain as high as 39.6% for the cement mortar and 32.5% for the cement paste specimens. Full crack closure was observed at 28 days for cement mortars with the healing agents. The self-healing system acted as a biological CO_3_^2−^ pump that can keep the bio-agents retained, protected, and active for up to 56 days of wet-dry incubation. This promising self-healing strategy requires further research and optimization.

## 1. Introduction

Concrete is the most popular construction material globally with 30 billion tons used annually in 80% of the construction cases [1,2]. Concrete can withstand compression but is vulnerable in tensile stress, which can lead to micro-cracks appearing as early as the setting process. Such cracks allow water, gases, and corrosive substances from the surrounding environment to ingress into the structure from an early stage and further diffusion through the interconnected porous network of the concrete matrix, causing structural degradation [3]. The limited flexural strength of concrete and diffusion of external substances through interconnected pores and cracks are the main factors in concrete degradation [3,4]. The degradation of concrete structures poses serious socioeconomic and environmental burdens, since cement production and distribution are responsible for 7% of global CO_2_ emissions [2], while the maintenance and repair of aging structures account for 30–50% of all spending in the construction sector [5]. Therefore, increasing the durability of concrete structures alongside lowering the maintenance costs and environmental impact of corrective measures have been the aims of multi-disciplinary research.

Conventional concrete remediation strategies such as cement replacement or the use of chemical fillers are limited to accessible and visible cracks; they are time-consuming and expensive as well. Therefore, research into self-healing systems for concrete structures has gained favor over the last two decades. Chemical and biological self-healing systems have since been devised and incorporated into different cement-based materials and tested for their self-healing ability [5,6,7,8,9,10,11,12]. Self-healing concrete broadly refers to cement-based materials that include an autogenous self-healing system. Biological concrete self-healing that provides an environmentally friendly self-healing option and can increase the service life of buildings and lower their maintenance cost has been gaining increasing research interest.

Biological concrete self-healing systems are based on the CaCO_3_ precipitation by various alkaliphilic bacteria through the microbially induced CaCO_3_ precipitation (MICP) process [5,7,13,14,15]. Bacterial CaCO_3_ can fill pores and micro-cracks that are formed during and after the concrete hardening process, thereby improving the structural integrity of the concrete and reducing the chance of degradation by the ingress of corrosives. CaCO_3_ is naturally precipitated in concrete through the reaction of atmospheric CO_2_ with Ca(OH)_2_, which is abundant in fresh concrete. CaCO_3_ thus produced in young concretes fills micro-cracks (0.01–0.1 μm) and pores, and the process is known as autogenous healing [16]. However, the occurrence of this phenomenon is largely limited to small cracks and fresh concretes with uncarbonated Ca(OH)_2_.

MICP can occur via various pathways, such as non-methylotrophic methanogenesis [17] and photosynthesis [18], and heterotrophic pathways such as ureolysis [4], conversion of organic salts [7,19,20], methane oxidation [21], sulfate reduction [22], and denitrification [23]. The pathways suitable for concrete self-healing are often referred to as ureolytic or non-ureolytic. Ureolytic MICP occurs through the generation of CO_3_^2−^ ions from urea hydrolysis, while non-ureolytic pathways generate CO_2_ through the metabolism of a range of precursor compounds. While different bacteria perform MICP through different metabolic pathways and at different rates, the MICP process is generally influenced by (1) Ca^2+^ concentration, (2) dissolved inorganic carbon concentration (DIC), (3) pH, and (4) nucleation site abundance [24,25,26,27].

To date, most of the bio-concrete studies have utilized ureolytic bacteria, which rely on the hydrolytic action of the urease enzyme on urea for CO_3_^2−^ production [28,29]. Urea hydrolysis is an efficient MICP pathway for the CaCO_3_ precipitation rate, and ureolytic bacteria and the enzyme urease are both well characterized [6]. NH_4_^+^ ions are released as a by-product of ureolytic MICP in a 1:1 molar ratio to precipitated CaCO_3._ The pH increase caused by NH_4_^+^ increases the CaCO_3_ precipitation rate in ureolytic self-healing concrete. However, if the NH_4_^+^ is in contact with nitrifying bacteria or conditions allow for its volatilization as NH_3(g)_, CaCO_3_ dissolution might occur following a drop in pH, which can be damaging for concrete structures [30]. In addition, NH_4_^+^ is an environmental pollutant that poses a significant risk to the health of different animal varieties, including humans [31]. Moreover, ureolytic bacteria are not ubiquitous, and they require specific conditions to survive and properly function [32]. Non-ureolytic MICP pathways have been considered environmentally friendly alternatives to ureolysis and have been researched for bio-concrete development. The oxidation of organic calcium salts such as calcium lactate (CaL), calcium formate, calcium acetate, CaCl_2_, calcium glutamate, and calcium nitrate are widespread among bacteria and are free of corrosive or polluting by-products [19,33,34]. This pathway is often described as an environmentally friendly alternative to ureolysis and has been investigated for applicability in concrete surface treatment and self-healing concrete research [19,33].

The direct addition of bacterial cells (vegetative cells or spores) and nutrients; i.e., healing agents, has been reported to adversely affect the compressive strength of concrete and severely reduce the survival of the cells due to high alkalinity and crushing, reducing the functionality of the self-healing system to only months [7,35]. Hence, researchers have attempted to immobilize the healing agents in different protective carriers instead of direct addition of the healing agents to concrete, since the immobilization of bacteria and nutrients holds significant advantages over the direct addition of healing components [36]. Expanded clay particles [16], low weight aggregate [37], and hydrogels [28] are examples of carriers used for self-healing concrete. Optimal protective materials should protect the healing agents until concrete faults such as crack formation allow water and oxygen to diffuse into the matrix and activate the self-healing system. Then, CaCO_3_ precipitation fills the crack and reinforces the structure.

Natural polysaccharides such as alginate and cellulose have been recently explored for the preparation of self-healing hydrogels due to desirable gelling properties, chemical stability, non-toxicity, economic advantages, and biodegradable nature [28,38,39,40]. Such natural polymers offer a sustainable pathway for the development of self-healing materials. Calcium alginate (CaAlg) capsules have been recently used as a protective material in self-healing concrete research [41]. Alginate is a hydrophilic, biodegradable polymer composed of mannuronic acid (M) and guluronic acid (G) monomers that are connected by glycosidic bonds and are commercially extracted from brown algae. Alginate monomers form homopolymeric (MM and GG) and heteropolymeric (MG) blocks. Alginate can form gels through complex formation with divalent cations such as Ca^2+^, which results in the cross-linking of alginate fibers, forming a porous CaAlg matrix. Important factors for the ionic gelation of alginate are temperature, alginate concentration, cation type, and concentration [42], while other parameters such as cross-linking duration, stirring rate, and alginate composition can also impact the gelation outcome. Commercially available alginate (as sodium alginate) can drastically vary regarding the proportion of MM, GG, and MG blocks, which can result in different chemical and physical properties. A recent report concluded that during cross-linking with Ca^2+^ ions, GG blocks are most readily cross-linked, followed by MG blocks [43]. MM blocks play a less significant role in CaAlg cross-linking.

The organic–inorganic network of CaAlg can absorb aqueous solutions many times its weight, landing CaAlg in the class of superabsorbent polymers (SAPs). Such polymers have been recently investigated for potential in concrete self-healing research, as they can trap bacterial spores, uptake water from the surrounding, and are eco-friendly and economically feasible [20,44,45]. Yet, they adversely affect the mechanical properties of cementitious materials as they may lead to increased porosity and reduced workability due to water uptake. Palin, Wiktor, and Jonkers [41] encapsulated alkaliphilic bacteria in CaAlg capsules for application in low-temperature marine environments and found that the system possessed great self-healing potential for concrete in contact with cold seawater. [44] assessed the impact of CaAlg on the compressive and flexural strength of concrete and found a 15% reduction in strength; i.e., it was much lower than other commercially available SAPs that can lower the concrete strength by up to 50%.

Since bacterial spores are the main agents behind biological concrete self-healing, determining the impact of the immobilization techniques on the bacterial population and the survival of the bacteria inside the cement environment are essential for ensuring the success of such self-healing treatment. In addition, accurate and exact survival determination can provide insight into the protective abilities of the capsule material and possibly the self-healing behavior the capsule offers (i.e., if healing agents are retained in the capsule or leached into the cement environment). While metabolic measurements such as the urea consumption (the TAN method) for ureolytic bacterial [28,45] and NO_3_^−^/NO_2_^−^ consumption measurements for non-ureolytic NO_3_^−^/NO_2_^−^ metabolizing bacteria [4,29,46,47,48,49,50] can be considered reliable measurements for bacterial survival and activity, while direct and accurate bacteria survival measurements are limited for non-ureolytic bacteria that do not metabolize NO_3_^−^/NO_2_^−^, e.g., those who oxidize organic salts. Since this class of non-ureolytic bacteria are the most prevalent in non-ureolytic concrete self-healing research, there is a need for better quantitative survival measurement strategies for such systems. The enumeration of spores has been used to estimate spore survival when the spores were directly added to cementitious materials by crushing the samples to free the spores [7,35], which is not suitable for immobilized or encapsulated spores. Hence, providing qualitative proof of spore survival has become a popular direction in this field [28,41,45,51,52]. This also includes survival determination methods such as O_2_ measurement and CaCO_3_ precipitation as proof of bacterial survival [16,41,53,54,55]. Methods such as O_2_ measurement suffer from disadvantages of indirect measures such as susceptibility to microbial contamination, which can go unnoticed; they can mainly distinguish between the presence and absence of bacteria, and it is not possible to know if the bacteria is still encapsulated or has leached out of the capsule. Therefore, there is a need for viability determination methods in concrete self-healing research that can give an exact measure of the bacterial viability and survival in each step of the work, from immobilization or encapsulation and throughout the in situ application phase. So far, the spore survival of non-ureolytic spores has only been directly measured when non-encapsulated spores were added directly to the cement samples by crushing the samples and enumerating the spores [7,35].

Here, we describe a procedure to quantitatively assess the survival of bacterial spores after CaAlg encapsulation. We further examined the spore retention ability of the CaAlg capsules during simulated cement mixing. Further in vitro characterizations were performed to assess the swelling of CaAlg capsules prepared with CaL or CaCl_2_. CaAlg is generally prepared with CaCl_2_, but the negative impact of Cl^−^ ions on concrete is a concern, while CaL has been shown to improve the early strength of concrete, and after alginate cross-linking, any remaining lactate can be consumed by the bacteria. Furthermore, the structural stability of the capsules was examined after exposure to conditions mimicking the extremes of concrete. The CaCO_3_ precipitation potential of the encapsulated spores was also verified. Then, CaAlg capsules were incorporated into cement paste and mortars, and the regain of flexural strength and crack healing by CaCO_3_ precipitation under wet–dry cycles were observed as self-healing hallmarks.

## 2. Materials and Methods

### 2.1. Materials

Pure culture of *Bacillus pseudofirmus* DSM 8715 was purchased from the German Collection of Microorganisms and Cell Cultures (DSMZ). *B. pseudofirmus* DSM 8715 is an alkalophilic aerobe isolated from lake-bed soil in Holstein, Germany. The optimal growth temperature and pH for *B. pseudofirmus* are 30 °C and 9.7, as suggested by DSMZ. *B. pseudofirmus* grows in the pH range from 7.5 to values exceeding 11.5 and has been reported to withstand oxidative stress in highly alkaline conditions [56]. *B. pseudofirmus* DSM 8715 colonies appeared as opaque-beige, flat-raised, and irregular on agar plates after 24 h of incubation, and colonies were 5–10 µm in length and about 1 µm in width. An overnight culture of *B. pseudofirmus* was stored in 25% (*v/v*) glycerol at −80 °C for further use.

Sodium alginate (Manugel GHB, FMC Biopolymer, Philadelphia, PA, USA) with the monomeric composition of 37% mannuronic acid (M) and 63% guluronic (G) was purchased and used for CaAlg capsule preparation. Ordinary portland cement (Type I) was used to prepare the cement paste and mortars.

### 2.2. Preparation Methods

#### 2.2.1. Growth Media Preparation

*B. pseudofirmus* was grown in 100 mL of nutrient broth (Merck, DE) containing 5 g/L peptone and 3 g/L meat extract adjusted to pH 9.7 using 1.0M NaOH in 250 mL Erlenmeyer flasks followed by sterilization by autoclaving at 121 °C for 15 min. The inoculated media was incubated at 30 °C overnight and stirred at 150 rpm. To induce CaCO_3_ precipitation, 90 mL of nutrient broth (pH 9.7) was supplemented with 10 mL of sterile 0.5 M CaL after autoclaving to achieve a final concentration of 0.05 M for CaL. The addition of CaL before autoclaving resulted in a cloudy media in contrast to the clear nutrient broth obtained when autoclaved CaL was added after autoclaving.

#### 2.2.2. Spore Suspension Preparation

To obtain spore suspension, a minimal basal salt media was adapted from a previous article [7]. First, 100 mL of the basal salt media was inoculated with an overnight culture of *B. pseudofirmus* in a 1% (*v/v*) ratio and incubated at 30 °C for 7 days. Spore levels were deduced by comparing colony-forming unit (CFU) estimations of the minimal basal salt media before and after pasteurization (85 °C, 15 min). Preliminary results indicated that the minimal basal salt media resulted in the full conversion of vegetative cells used to inoculate the media to spores. Spores were isolated by centrifugation (9300× *g*, 7 min) and washed 3 times with sterile water. Then, the resulting spore paste was resuspended in 2 mL of autoclaved distilled water (DW) and stored at 4 °C for further use.

#### 2.2.3. Encapsulation of Nutrients and Spores into the CaAlg

**Figure 1:**
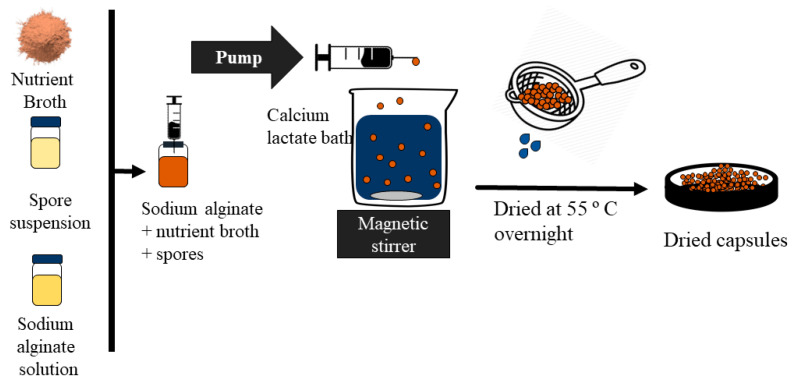
Encapsulation procedure for NS1–CaAlg capsules.

The encapsulation technique used in this project is based on the ionic gelation of alginate upon contact with Ca^2+^ ions (Figure 1). This procedure was used for the preparation of O, N, S, NS1, and NS2 capsules, following the composition described in Table 1. The two capsules types containing all the required healing agents, NS1 and NS2, only differ in spore concentration, which was set to a higher amount in NS2 capsules to examine the effect of this factor on concrete self-healing. The encapsulation solution composed of sodium alginate (2% *w/v*) and nutrient broth (8 g/L) was fully dissolved in DW and autoclaved at 121 °C for 15 min. Then, the autoclaved alginate–broth mixture was left to cool overnight. Then, the spore suspension was used to seed the encapsulation solution following a fixed 2.5% *v/v* ratio. The mixture was then added dropwise to 200 mL of a sterile 0.20 M CaL bath in a 250 mL glass beaker using a 12 mL syringe from the fixed height of 8 cm using a syringe pump. The flow rate of the syringe pump was adjusted to 90 mL/h. The CaL bath was continuously stirred using a magnetic stirrer (100 rpm). CaAlg capsules were allowed to stir in the bath for 20 min for full CaAlg cross-linking. The same procedure was used for the capsules prepared using CaCl_2_.

Then, the capsules were separated from the CaL bath using a sieve, washed with autoclaved water, placed in sterile Petri dishes, and dried in the oven at 55 °C overnight. The parameters for CaAlg encapsulation have been taken from a previous study [41]. The distance between the needle and the CaL bath was adjusted through trial and error and maintained at 8 cm.

#### 2.2.4. Test Solution Preparation

Capsules were resuspended in the following solutions for analysis: (1) DW (pH 8 ± 1), (2) 10% cement filtrate (CF) (pH 12 ± 1). 10% CF was prepared by mixing 10 g of cement in 100 mL of DW for 24 h. Then, the solution was filtered using a filter paper with a pore size of <0.2 µm. All test solutions were places in 250 mL flasks and sterilized by autoclaving.

#### 2.2.5. Preparation of Cement Paste and Mortars for Flexural Analysis

**Figure 2:**
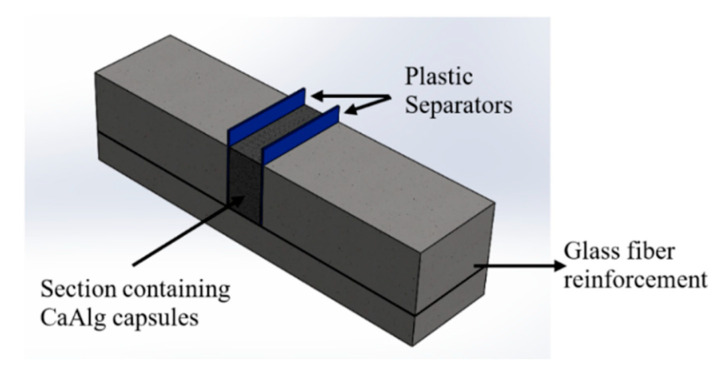
Schematic of the mold separation to incorporate the CaAlg capsules to the middle section.

**Figure 3:**
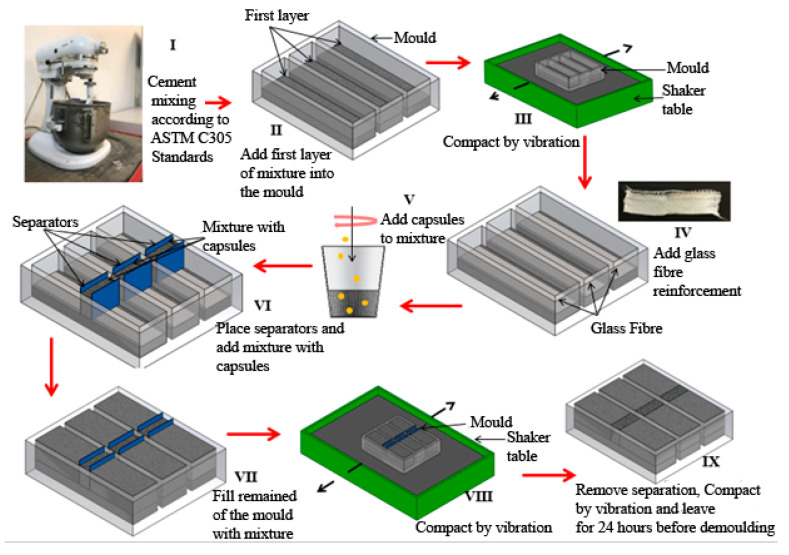
Preparation procedure for the cement paste and mortars containing CaAlg capsules.

The cement paste and mortar samples were prepared according to the ASTM C305 standards. A water-to-cement (ordinary Portland cement) ratio of 0.35 was used in the preparation of the cement mortars. Cement mortars were prepared with a sand-to-cement ratio of 1 and water-to-cement ratio of 0.35. A mold with dimensions of 25 mm × 25 mm × 100 mm was used to prepare the cement paste and mortars. Initially, one-third of the specimen was filled with the mixture and compacted through vibration 5–6 times in 30-second intervals. A layer of the glass fiber was placed on the initial layer to avoid complete mortar cracking for subsequent self-healing observations. The capsules were only added to the middle section of the samples (above the initial layer). The sections in the mold were separated using thin plastic separators (Figure 2). The middle section of the mold was filled with a paste consisting of 5% CaAlg capsules by volume of the sample, and the two sides were filled with paste without capsules. Each sample consisted of 202 mg of capsules, which amounted to approximately 74 capsules. Then, the samples were vibrated 5–6 times at 30-s intervals before and after removing the separations and allowed to rest for 24 h before de-molding. A summary of the sample preparation is provided in Table 2. Triplicate samples were observed after 28 and 56 days of self-healing. The step-by-step process of sample preparation is depicted in Figure 3.

### 2.3. In Vitro Characterization Methods

#### 2.3.1. Survival of Encapsulated Bacterial Spores

The survival of encapsulated spores was quantified by dissolving 5 S or NS1 capsules in 5 mL of sterile citrate buffer (55 mM trisodium citrate, 30 mM anhydrous EDTA, 150 mM NaCl, pH 8) for 30 min at 150 rpm. Serial dilution and CFU estimation of dissolved capsule solution were used to estimate the survival of encapsulated spores. First, 100 µL mL of the suspension was serially diluted in sterile 0.85% saline solution for CFU estimation by spread plating on nutrient agar plates (pH 9.7). Colony count was obtained after 48 h incubation at 30 °C. N capsules were tested as negative control. This test was done on 3 biological replicates.

#### 2.3.2. Swelling and Stability of CaAlg Capsules

The swelling assessment was done using N capsules cross-linked with either CaCl_2_ or CaL, which were resuspended in CF or DW for 14 days. The volume of the capsules was measured before and after suspension using a Mitutoyo 293-130-10 High Accuracy Digimatic Micrometer, and percentage swelling was determined using Equation (1):

[(Volume after suspension–Volume before suspension)/Volume before suspension] × 100.(1)

The stability of the two capsule types in the test solutions was studied by FE-SEM imaging after the 14-day test period. Capsules were removed and washed. Diameters were measured, and then the capsules were dried before for morphological analysis. Some capsules were cut in half before drying to observe the cross-section.

#### 2.3.3. Nutrient Leaching in CaAlg Capsules

UV absorption at 280 nm wavelength was used to examine the leaching of nutrient broth from N capsules prepared with CaL or CaCl_2_ with a Tecan Infinite M200 microplate reader (Austria), based on the absorption of UV radiation at 280 nm by aromatic proteins. Ten capsules were suspended in 1 mL of CF and measured after 1, 2, and 14 days in separate solutions. Then, 100 µL aliquots were taken from the suspension after selected time points. O capsules prepared with either CaL or CaCl_2_ were used as controls, and the absorbance values for their solutions were used to blank the corresponding N solutions. This test was done in biological triplicates.

#### 2.3.4. Spore Retention of CaAlg Capsules

To measure the spore retention ability of S and NS1 capsules, 5 capsules of each type were resuspended in 5 mL of DW or CF and intensely vortexed for 2 min. Then, 100 µL of the suspension was serially diluted in sterile 0.85% saline solution for CFU estimation. Dilutions were spread on nutrient agar (pH 9.7) plates. Colony count was obtained after 48 h incubation at 30 °C. N capsules were tested as negative control. This test was done on 3 biological replicates.

To estimate the spore retention ability of crushed S and NS1 capsules, 5 capsules of each type were crushed to powder using a mortar and pestle. Then, crushed capsules were resuspended in DW or CF and intensely vortexed for 2 min. Afterwards, 100 µL of the mixture was serially diluted in sterile 0.85% saline solution for CFU estimation. Dilutions were spread on nutrient agar (pH 9.7) plates. Colony count was obtained after 48 h incubation at 30 °C. N capsules were tested as negative control. This test was done on 3 biological replicates.

#### 2.3.5. CaCO_3_ Precipitation by Encapsulated Bacterial Spores

To assess if immobilized bacteria precipitate CaCO_3_, NS1 capsules were resuspended in DW or CF or placed inside cement samples, which were cured underwater for 2 weeks. O, N, and S capsules were tested as negative controls. Ten capsules were resuspended in 100 mL of DW or CF in a 250 mL flask and then incubated at 25 °C without shaking. After 7 days, capsules were filtered out of the test solutions and thoroughly washed. Then, capsules were examined under an optical microscope for the presence of crystalline precipitates and subjected to chemical analysis.

Alongside capsule suspension in test solutions, capsules were also placed inside small cement samples and placed in DW for 14 days. To obtain cement paste, Portland cement was used to prepare cement paste following the water/cement 0.35 weight ratio. O, S, N, and NS1 capsules were added to designated samples by mixing the capsuled with cement before hydration. Silicon molds (9 × 1 × 0.75 cm) were used for casting the cement samples. The samples were kept inside the mold at room temperature for 24 h. After de-molding, cement samples were cured in DW for 14 days at room temperature without capping the containers or shaking. Capsules were monitored during the incubation period through morphological analysis.

#### 2.3.6. Statistical Analysis

One-way ANOVA was used to determine if the survival of S and NS1 capsules significantly differed from each other or the expected survival estimation calculated in S1 (*p* < 0.05). One-way ANOVA was also used to compare the difference in spore retention of the capsules. Tukey’s post-hoc test was also conducted for multiple comparisons between the determined spore survival in each capsule type and the theoretical survival value. The results of this analysis are shown in Figure S1.

#### 2.3.7. Fourier Transform Infrared Spectroscopy (FTIR) Analysis

FTIR spectroscopy (Spectrum 100, Perkin-Elmer Inc., Waltham, MA, USA) was conducted to determine if CaCO_3_ precipitation occurred in NS1 and negative control capsules resuspended in CF or DW for 7 days. Capsules were dried at 55 °C and ground to powder. Spectra were the result of 16 scans in the range of 4000–600 cm^−1^ with a resolution of 2 cm^−1^.

CaCO_3_ has 3 main polymorphs with different FTIR peaks: v3 corresponding to an asymmetric C–O stretching vibration, v2 corresponding to an out-of-plane bending vibration, and v4 corresponding to a planer bending vibration of the C–O bond.

#### 2.3.8. Thermogravimetric Analysis (TGA) of Capsules

TGA (Q50, TA Instruments, New Castle, DE, USA) was conducted on S and NS1 capsules resuspended in CF for 1 week. TGA recorded a change in the weight of the sample as the temperature increased at 10 °C/min from room temperature to over 895 °C in an N_2_ atmosphere. As CaCO_3_ is decomposed to CO_2_ and CO_3_^2−^ in the 650–800 °C temperature range; changes in sample weight at this range can be attributed to CaCO_3_ presence in/on the samples [57].

### 2.4. In Situ Self-Healing Efficiency Assessment of Cement Paste and Mortars

#### 2.4.1. Flexural Testing

Cracks were initiated in the reinforced, flexural cement paste and mortar specimen via a three-point bending test using an Instron 5966 testing rig (Instron, Norwood, MA, USA) after being cured in DW for 7 days (referred from hereon as Day 0) at room temperature. The uniform stress in a three-point bending test is concentrated in a small area where the load is applied. The section containing the capsules are in tension during the test. A single crack is initiated in the section containing the CaAlg capsules due to them being under the reinforced design of the flexural samples. The layer of glass fiber impedes the crack propagation, which prevents the sample from separating into two parts. The load was applied to the samples at a rate of 0.2 mm/min until a crack width of approximately 1 mm was achieved. The crack shrinks to an approximate width of 0.1–0.3 mm after the load is removed. After the crack initiation, the samples were subjected to 12-h wet and dry cycles to more realistically mimic the environment for the self-healing of cracks via CaCO_3_ precipitation. Flexural tests were performed on the samples after 28 and 56 days of being subjected to wet and dry cycles to quantify the effect that the crack closure due to MICP has on the flexural strength of the samples. The healing efficiency of the different treatments was deduced by comparing the flexural strength of the samples after crack initiation to the flexural strength recorded after 28 and 56 days of wet–dry incubation.

#### 2.4.2. Crack Width Measurements and Visualization of Self-Healing

The cracks formed in the flexural samples were documented before and after self-healing. The cracks were observed through an MPlan N 5×/0.10∞/- OLYMPUS microscope objective lens (OLYMPUS, Tokyo, Japan) and a Nikon DS-Fi1 microscope. The software used to measure the crack widths of the samples was NIS-Elements D3.1. The crack widths were measured from several places along the crack on the surface of the samples, and an average crack width was calculated. A preliminary visualization of the cracks before and after self-healing was documented using a 12-megapixel camera lens with a 5× zoom.

#### 2.4.3. Morphological Analysis

HITACHI SU 8010 Field Emission Scanning Electron Microscope (FE-SEM) (HITACHI, Tokyo, Japan) was used to analyze the morphology of the precipitates and capsules in the in vitro tests and to obtain a clear visualization of the behavior of the CaAlg capsules after crack initiation and to observe the morphology of the precipitates in the cement paste and mortars. Conductive carbon tape was placed on the sample holder before placing the samples. Then, the samples were coated with platinum by sputtering to increase the conductivity of the samples. An accelerating voltage between 3 and 5 kV was used. SEM images were taken from capsules exposed to the cement environment in vitro, as well as from capsules placed in cement paste and mortars after self-healing.

## 3. Results and Discussion

### 3.1. Survival of Encapsulated Bacterial Spores

Alginate capsules were subjected to de-cross-linking in a citrate–EDTA buffer to estimate the survival of the encapsulated spores in S, NS1, and NS2 capsules (Table 3). The obtained values were compared to the expected results based on the initial spore concentration in the encapsulation solution of each capsule (S1).

One-way ANOVA results (Figure S1) showed no significant difference either between the spore survival of S and NS1 capsules or between the calculated theoretical and experimental survival values for each capsule type. Hence, it can be assumed that CaAlg encapsulation does not impact the survival of bacterial spores, and that the presence of nutrients in the capsules did not impact the encapsulation process nor the survival test procedure. N capsules were assessed as negative control and contained no bacteria. No contaminating colonies were observed from the dissolution of any capsules in the citrate–EDTA buffer.

Previous studies on biological self-healing concrete that employed a carrier material for bacteria immobilization have largely reported proof of survival or indirectly measured bacterial [28,41,45,51,52]. Since the quantity of CaCO_3_ formed through MICP is a direct function of the levels of bacterial activity [51], it is essential to determine if the encapsulation process affects the survival of the encapsulated bacteria. The quantification of spore survival can also provide insights into the degree of environmental protection that the carrier material provides for the encapsulated spores. As our findings show that CaAlg encapsulation did not impact the survival of the encapsulated *B. pseudofirmus* spores, it can be deduced that CaAlg is a biocompatible capsule for *B. pseudofirmus* spores. The lack of any significant difference between the survival of S and NS1 capsules indicates that the presence of nutrients in the capsules did not impact the encapsulation process or spore survival (Figure S1). Since the survival of encapsulated spores was determined by preparing and dissolving biological replicate capsules, the variations in survival results reflect the entire process: from spore growth and harvest to capsule drying and their subsequent dissolution for survival determination. Since only small variations were found in survival results (Table 3), the entire process of capsule preparation is hence reliable and consistent.

The absence of any contaminating colonies and lack of any growth when N capsules were assessed for spore survival suggest that the encapsulation process does not introduce unwanted, contaminating microbes into the capsules. Although the incubation period for the CFU estimation test was 48 h, the majority of colonies appeared after 24 h, suggesting that spore germination was not delayed. A mixture of Na-citrate and EDTA was used to dissolve CaAlg capsules for survival measurement. Na-citrate and EDTA de-cross-linked CaAlg by chelating Ca^2+^ bond to alginate fibers [58]. Our preliminary investigation indicated that the citrate–EDTA buffer had no impact on the survival of free spores, although EDTA has been reported to negatively impact spore survival under specific conditions by chelating metal cations from the spore coat [59]. This may have been due to the relatively low EDTA concentration and the chelation of de-cross-linked Ca^2+^ by EDTA, lowering the chance of any impact by EDTA on the released spores. Our primary assessments also showed that spore loss during capsule preparation, specifically at the CaL bath stage, was below the detection limit. This adheres to the survival results of encapsulated spores, which indicate little to no loss of spores throughout the encapsulation process.

Direct survival determination for encapsulated bacteria holds advantages over indirect methods previously used, such as determining the activity of enzyme urease by measuring changes in NH_3_ concentration when immobilized bacteria are placed in a urea-rich media. The urease method is only applicable when immobilized bacteria are ureolytic and cannot be used to deduce the survival of bacteria that conduct non-ureolytic MICP. This method has been previously employed to estimate the survival of immobilized ureolytic bacteria and reported higher urea decomposition when immobilized spores were placed in a urea-rich medium compared to when the spore-free carrier was placed in the urea-rich media [4,29]. Although these findings serve as proof of survival for encapsulated spores, they do not offer insight into the impact of immobilization on spore survival. In addition, these studies suggested that autoclaving and contamination due to non-sterility of the carrier material might have led to urea degradation in negative control samples that lacked spores. The direct evaluation method used in our investigation allows the identification of contaminants based on colony morphology and staining procedures. We observed no growth in negative control samples (N capsules), no notable contamination in NS1 and NS2 capsules, and hence determined the survival of the immobilized bacteria directly. Although researchers have resuspended crushed capsules in urea-rich media, there is no way of knowing the true degree of spore germination or if contamination by other microbes is interfering with the urea decomposition rate [45]. Since direct survival estimation can demonstrate spore germination, colony growth, the presence of contaminating microbes, and can be used to quantify the survival of any self-healing bacteria (e.g., ureolytic and non-ureolytic), direct survival evaluation holds important advantages over indirect survival assessment.

### 3.2. Swelling and Stability of the CaAlg Capsules

**Figure 4:**
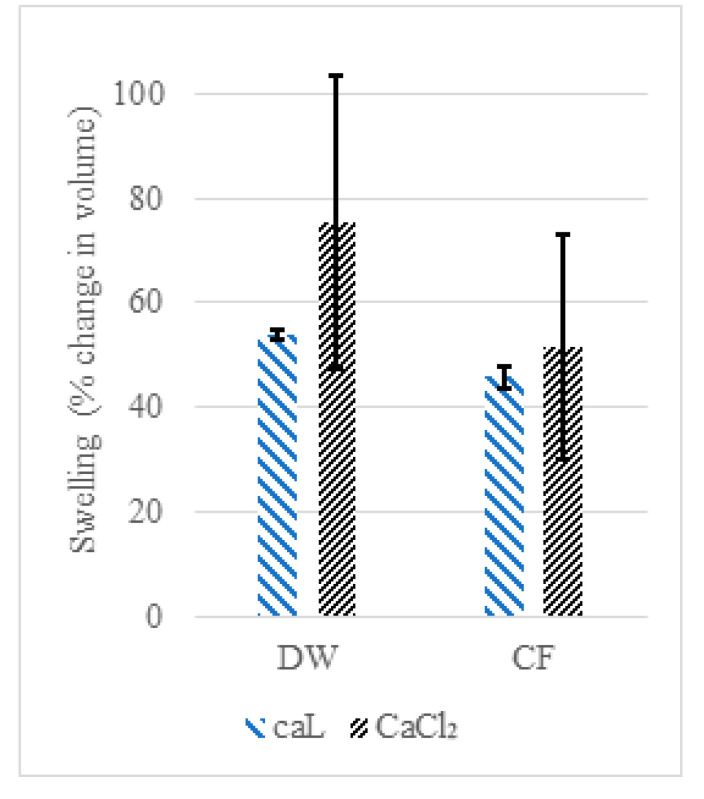
Swelling of CaAlg capsuled prepared using either CaL or CaCl_2_ in water or CF after 14 days.

**Figure 5:**
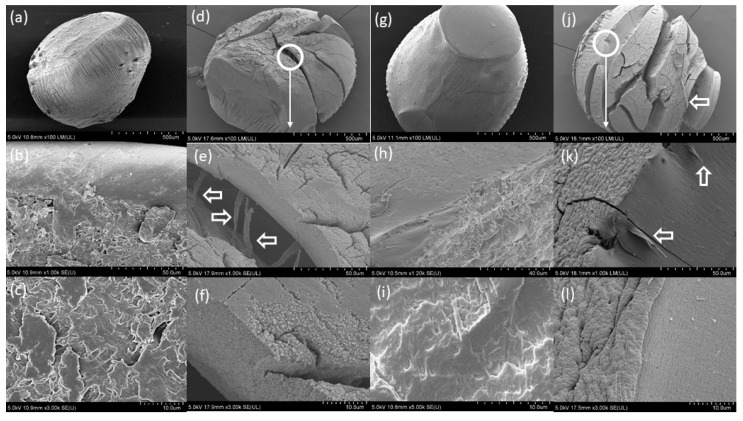
FE-SEM micrographs of N capsules prepared: (**a**) Pristine CaAlg capsules prepared with CaCl_2_ (**b**) cross-section of pristine CaAlg capsules prepared with CaCl_2_; (**c**) hydrogel matrix of pristine CaAlg capsules prepared with CaCl_2_. (**d**–**f**) CaAlg capsules prepared with CaCl_2_ after suspension in CF for 14 days; (**g**) pristine CaAlg capsules prepared with calcium lactate (CaL); (**h**) cross-section of pristine CaAlg capsules prepared with CaL; (**i**) hydrogel matrix of pristine CaAlg capsules prepared with CaL; (**j**–**l**) CaAlg capsules prepared with CaL after suspension in cement filtrate (CF) for 14 days.

Capsules prepared with CaL were on average 26.2 ± 2% larger in volume than capsules prepared with CaCl_2_ after the initial drying. Fourteen-day swelling results of the N capsules prepared using CaL or CaCl_2_ showed no difference between either group when suspended in DW or CF (Figure 4), with the CaCl_2_ series capsules suspended in DW showing the highest swelling of 76% on average. CaCl_2_ series capsules suspended in DW or CF both exhibited more than 90% size deviation compared to CaL series capsules, which were more uniform in size. FE-SEM analysis of the capsules from both the CaCl_2_ and CaL series suspended in CF exhibited large cracks and lamination (Figure 5d,e,j,k) and fibrous extrusions (Figure 5e,j,k, white arrows) signaling polymer degradation. The outer surface of the capsules from both the CaCl_2_ and CaL series had morphologically changed (Figure 5e,f,k,l), with the surface of the CaCl_2_ series capsule showing signs of degradation and possible precipitation (Figure 5f). The cracks no longer exhibited the matrix morphology of the pristine capsules (Figure 5c,f,i,k,l). The previous comparison between CaAlg capsules prepared with either CaL or CaCl_2_ reported faster gelation with CaCl_2_ [60] possibly due to the higher solubility of CaCl_2_ (75 g/L) compared to CaL (8 g/L), which results in faster Ca^2+^–Alg interactions, but both Ca sources eventually achieve the same gel strength and structure.

To the best of our knowledge, no reports of CaAlg degradation due to alkalinity have been made to date. However, there is evidence of cation exchange in the Ca–M block of alginate under salt attack [61] or in the presence of other cations [62], which may explain the morphological observations. CF is highly alkaline and ionic and can result in salt attacks on the CaAlg structure. The larger variation in the swelling of the CaCl_2_ series compared to the CaL series requires further examination, as the available literature on this particular observation is lacking. This matter can be subjected to future research by using a larger sample size with capsules of consistent dimensions.

The dry, untreated capsules of the CaCl_2_ series were as homogeneous in size as the CaL series capsules, and the swelling variation can only be due to the solution suspension effects.

### 3.3. Spore Retention Ability of the CaAlg Capsules

No bacterial colonies were observed for either capsule type in DW or CF, for intact or crushed capsules. This lack of bacterial detection points to the excellent spore-retention ability of the CaAlg capsules, showing that almost all spores remain trapped between cross-linked alginate chains, even when the CaAlg matrix is shattered. In addition, the spore retention ability of CaAlg capsules is not altered by the cement environment, as simulated using CF, or the presence of nutrients in the capsules.

To understand the behavior of capsules in the cement environment, it is essential to determine if the capsules retain the encapsulated spores when applied in situ or if the spores leach out of the capsules and to what degree. Spore loss from CaAlg capsules could occur during concrete preparation, which exposes the capsules to extreme alkalinity and physical shear. Our findings show that intact CaAlg capsules retained almost all encapsulated spores regardless of the test solution used. This suggests that little to no spores would leach into fresh cement during the cement mixing process. This observation was also made by testing the spore loss from crushed capsules. Therefore, the encapsulated spores most likely contribute to self-healing by providing CO_3_^2−^ ions needed for CaCO_3_ precipitation, and they do not directly act as nucleation sites for crystal formation, even when CaAlg capsules are structurally compromised. However, our findings offer no clues about the spore retention of capsules exposed to the cement environment for extended periods, which should be evaluated in future studies alongside the changes to the CaAlg structure, if any.

### 3.4. Nutrient Retention of CaAlg Capsules

**Figure 6:**
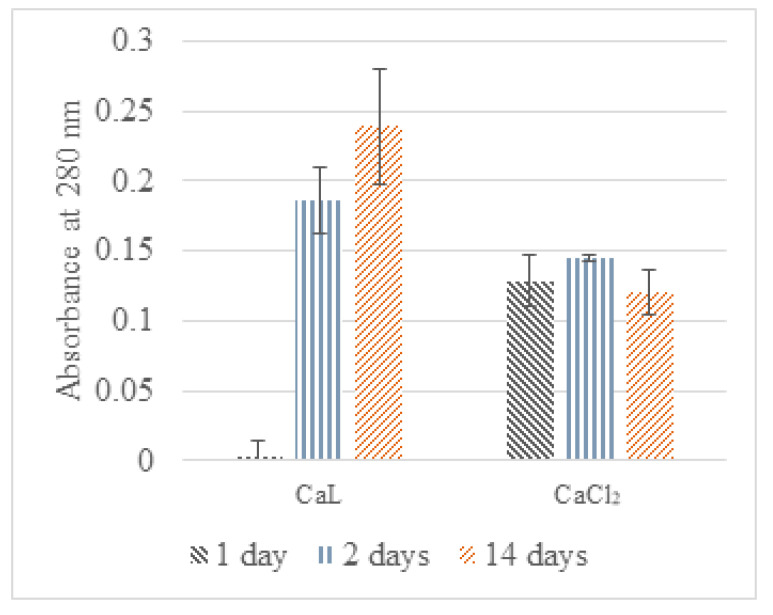
UV absorbance results (280 nm) of CF solutions with suspended N capsules from the CaL and the CaCl_2_ series for 1, 2, and 14 days.

UV absorbance results of DW and CF solutions containing N capsules from either the CaL series or the CaCl_2_ series differ significantly between the two capsule types and the time points (Figure 6). For CaL capsules, the absorbance differs significantly between day 1 and days 2 and 14, while there is no significant difference between the absorbance of different time points for the CaCl_2_ capsules. Since nutrient broth is expected to leach out of the capsules by diffusion [63], the leaching would stop once the concentration of the nutrient broth in the solution is in equilibrium to that of the capsules. This would suggest that for the CaCl_2_ capsules, the equilibrium was reached on the first day in both DW and CF. For the CaL capsules, this concentration was reached on the second day for DW and likely after the second day in the CF. Hence, it can be assumed that CaL capsules would leach fewer nutrients in short-term exposure to DW compared to CaCl_2_ capsules but more after prolonged exposure.

The nutrient broth is composed of water-soluble components such as glucose, small peptides, short-chain amino acids, and singular amino acids. Therefore, porous hydrogels such as CaAlg capsules are unlikely to retain nutrients for long if directly exposed to DW, as nutrients are expected to leach out. Yet, the bacteria used in this research, *B. pseudofirmus,* can precipitate CaCO_3_ even when nutrients are scarce, and hence nutrient leaching from such capsules is not a good indicator for their applicability in this field. However, nutrient leaching would limit their applicability for long-term self-healing involving more than once self-healing cycle and calls for further improvement to hydrogel-based self-healing systems if long-term, cyclic self-healing performance is the desired performance of the system. Based on previous research on the diffusion of encapsulated biological molecules such as amino acids and proteins from CaAlg [64], it can be expected that smaller molecules such as singular amino acids are likely to leach early, while larger or heavier molecules such as peptides may leach out later. At this step, the nutrient broth leached out of the capsules was not quantified due to multiple interfering factors such as the possibility of salt–amino acid or salt–peptide complex formations, which can easily alter the UV absorbance reading [65,66]. However, the in situ self-healing performance of the capsules (Section 3.6.2) would shed more light on the nutrient retention ability of the capsules. There is a need for accurate and concise methods to study nutrient retention in the cement environment, which can be addressed in future studies.

### 3.5. CaCO_3_ Precipitation by Encapsulated Spores

**Figure 7:**
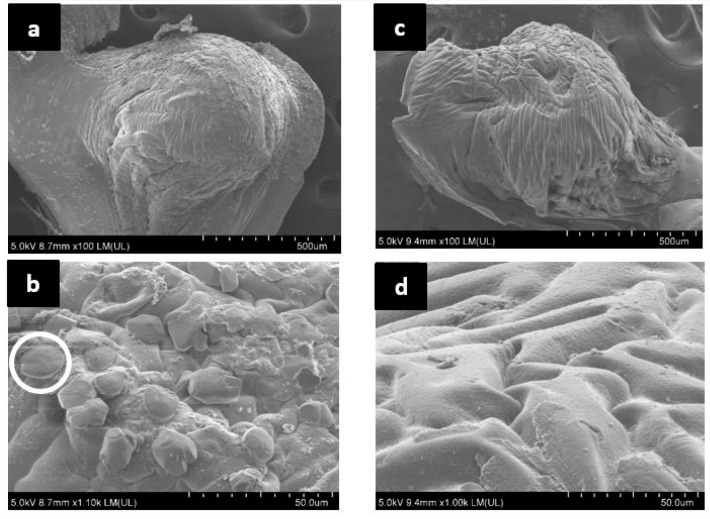
FE-SEM micrographs of S and NS1 capsules resuspended in CF for 7 days. (**a**) NS1 capsule after 7 days of resuspension in CF ×100 magnification (**b**) NS1 capsule after 7 days of resuspension in CF ×1000 magnification (**c**) S capsule after 7 days of resuspension in CF ×100 magnification (**d**) S capsule after 7 days of resuspension in CF ×1000 magnification.

**Figure 8:**
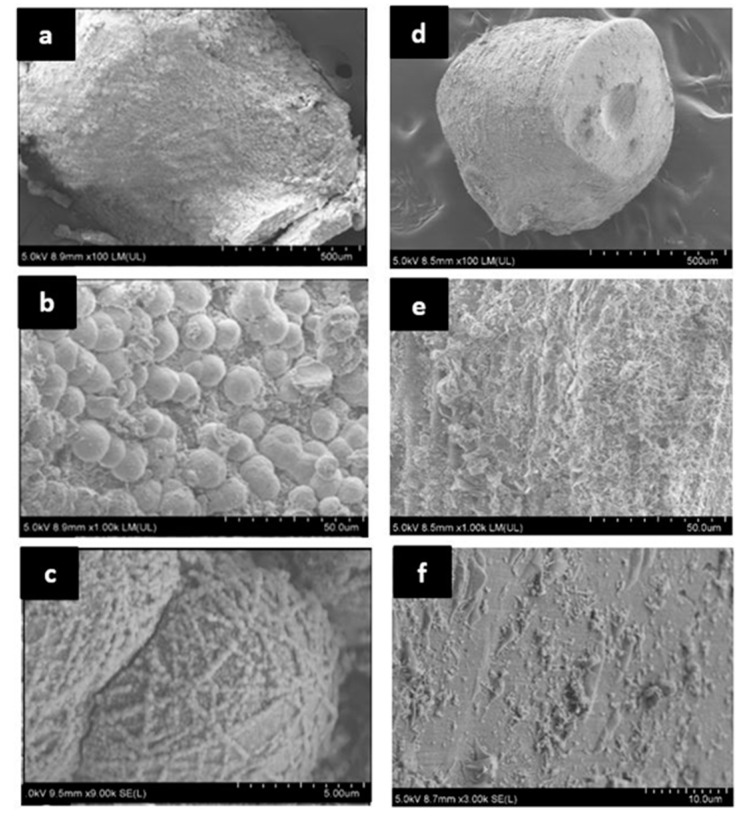
FE-SEM micrographs of NS1 and O capsules placed in cement paste samples and cured in distilled water (DW) for 14 days. (**a**) NS1 capsule after 14 days inside cement ×100 magnification; (**b**) NS1 capsule after 14 days inside cement ×1000 magnification; (**c**) NS1 capsule after 14 days inside cement > ×1000 magnification; (**d**) O capsule after 14 days inside cement ×100 magnification; (**e**) O capsule after 14 days inside cement ×1000 magnification; (**f**) O capsule after 14 days inside cement > ×1000 magnification.

Precipitation was microscopically observed on the surface of NS1 capsules resuspended in CF (Figure 7) and all the capsules placed inside cement paste samples (Figure 8). Precipitates appeared as crystals, either as individual grains or as chains or clusters. FE-SEM analysis of NS1 capsules resuspended in CF (Figure 7a,b, white circle) showed semi-spherical precipitates on the capsule surface or extruding from the capsule boundary, with an approximate diameter of 10 µm (Figure 7b, white circle). Precipitates were not evenly distributed across the surface of NS1 capsules (Figure 7a). No evidence of precipitates was observed on NS1 capsules resuspended in DW (Figure S2). Negative control capsules O and N presented no evidence of precipitation after resuspension in either test solution. FE-SEM analysis of the surface of S capsules resuspended in CF is shown as a representative of negative control capsules and includes a surface clear of precipitates (Figure 7c,d).

**Figure 9:**
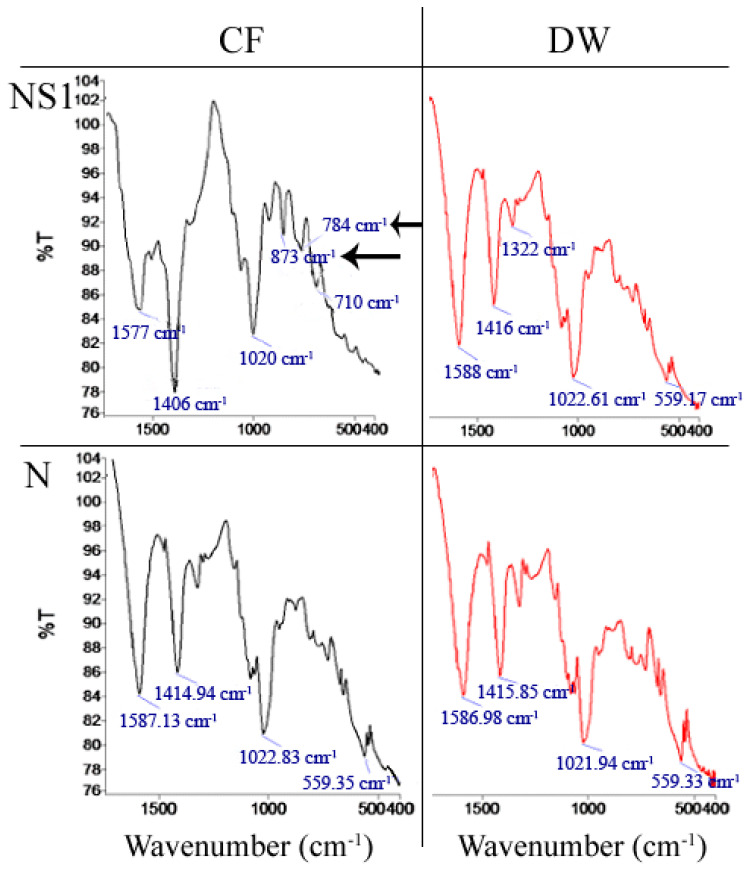
Fourier Transform Infrared (FTIR) spectra of NS1 and N capsules resuspended in CF or DW.

**Figure 10:**
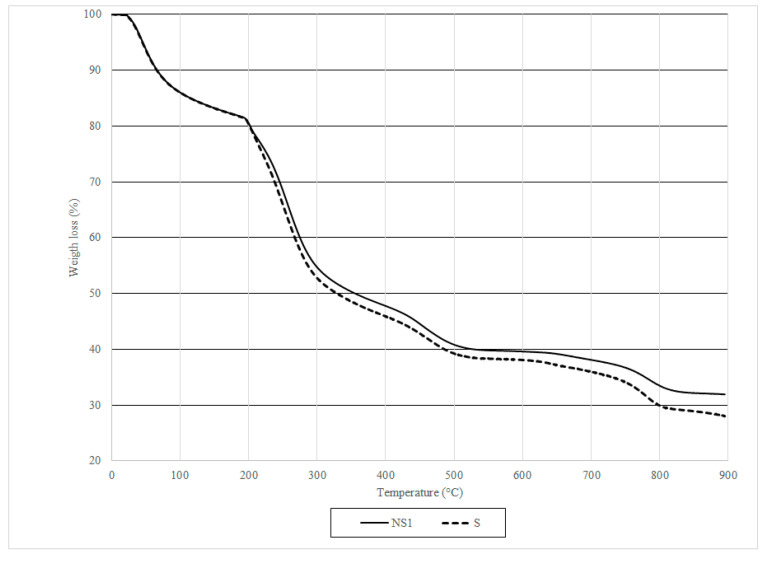
Thermogravimetric analysis (TGA) results of NS1 and Scapsules after 7 days in CF. Solid line: NS1 capsules, dashed line: S capsules.

FTIR analysis suggests that CaCO_3_ is present in NS1 capsules that were incorporated in the CF (Figure 9) and absent from negative control capsule N resuspended in CF (Figure 9). TGA results confirm the presence of CaCO_3_ in NS1 capsules resuspended in CF (Figure 10). However, as CaCO_3_ may be sourced from the CF and not be of biological origin, TGA was also performed on N capsules resuspended in CF. TGA results show somewhat lower weight loss for NS1 capsules than N capsules after 7 days in CF after heating to 895 °C (Figure 10). This observation, coupled with FTIR analysis and FE-SEM imaging, serves as a proof of CaCO_3_ precipitation by *B. pseudofirmus* encapsulated in CaAlg capsules.

As expected, CaCO_3_ precipitates were only observed in NS1 capsules resuspended in CF in the presence of spores and calcium under alkaline conditions. This observation is reinforced by results of FTIR analysis (Figure 9) and TGA (for NS1 capsules only, Figure 10), and coupled with the absence of precipitation in NS1 and negative control capsules resuspended in DW (Figure 7 and Figure 8, Figure S2), it serves as a proof of CaCO_3_ precipitation by CaAlg-encapsulated *B. pseudofirmus*. Based on our findings and previous reports [41], CaAlg-encapsulated spores germinate as the result of water uptake by the porous CaAlg capsules, which leads to the availability of nutrients and oxygen to spores, leading to spore germination inside CaAlg capsules. This suggests that CaAlg-immobilized spores do not require capsules breakage to germinate. This hypothesis is reinforced by spore retention results and can be further tested by observing the behavior of capsules placed in cement.

Although our findings point to the successful germination of CaAlg-encapsulated spores, it is not known if all the encapsulated spores germinate in intact capsules. CaCO_3_ precipitation in capsule NS1 could only be explained by the absorption of Ca^2+^ from the cement solution, as no Ca^2+^ was originally present in the capsules. The porous nature of alginate allows CO_3_^2−^, which results from the metabolism of nutrients, to diffuse out of the capsule. Our observations suggest that the capsule surface acts as the nucleation site for CaCO_3_ formation, providing the energy and surface area needed for precipitation. This adheres to findings by [41], who reported the majority of precipitates at the CaAlg capsule surface. However, observation of precipitation only on the capsule surface may be due to the germination of spores closer to the capsule parameter rather than spores embedded deep into the CaAlg matrix. A previous study found the effective oxygen penetration range to be 0.1–0.15 mm in CaAlg capsules [67]. Although *B. pseudofirmus* is a facultative anaerobe, higher oxygen levels are needed for spore germination than for CaCO_3_ precipitation by a vegetative cell. Spore distribution in CaAlg capsules and spatial patterns of spore germination for trapped spores warrant further studies.

The absence of any distinguishable precipitates on the surface of negative control capsules resuspended in cement solution may be explained by the fact that the bottles which contained capsules and CF were capped tightly, keeping atmospheric CO_2_ from entering the bottle and resulting in CaCO_3_ precipitation. CaCO_3_ precipitates in capsules of either type following 7 days of resuspension in DW is as expected, since DW does not provide the alkaline environment needed for the germination and growth of *B. pseudofirmus* spores, and no calcium source was available.

The presence of biological precipitates in treatment capsules and lack thereof in negative control samples after 14 days of curing in DW is expected (Figure 8, Figure S2), based on the precipitation results from treatment capsules resuspended in CF. Unlike the negative control capsules resuspended in CF, negative control capsules placed in cement exhibited particles and precipitates on their surface (Figure S2). As mineral precipitation naturally occurs during cement curing, negative control capsules may have acted as nucleation sites for precipitation and facilitated mineral formation. Hence, our microscopic observations cannot offer any definitive conclusions on how the precipitates formed, i.e., if the precipitates formed through the reaction of Ca(OH)_2_ native to cement with atmospheric CO_2_, or through the reaction with CO_3_^2−^ resulting from bacterial metabolism. However, a clear difference could be seen in the number of precipitates that had formed on the NS1 capsule surface compared to negative control capsules (Figure 8, Figure S2). Precipitates observed on the surface of NS1 capsules (Figure 8b) adhere to previous observations of CaCO_3_ crystals formed in alginate capsules with regard to size and shape [41].

The difference observed between NS1 capsules that were directly or partially exposed to water to capsules fully encased in cement reinforces the hypothesis that CaAlg-encapsulated spores do not require capsule breakage, but they do require exposure to air and water for germination, CO_3_^2−^ release from the CaAlg capsules, and subsequent CaCO_3_ precipitation in the cement environment. This observation points to CaAlg capsules as promising protective carriers for concrete self-healing, since CaAlg capsules keep spores dormant until direct exposure to environmental elements. With regard to self-healing concrete, CaAlg capsules not on/near the surface would only be directly exposed to water and air if cracks allow their diffusion into the structure. Hence, crack formation would likely be the main trigger for the concrete self-healing ability of CaAlg capsules when applied in situ.

### 3.6. Flexural Strength Results of Cement Paste and Mortars

#### 3.6.1. Flexural Strength of Cement Paste and Mortars before Self-Healing

**Figure 11:**
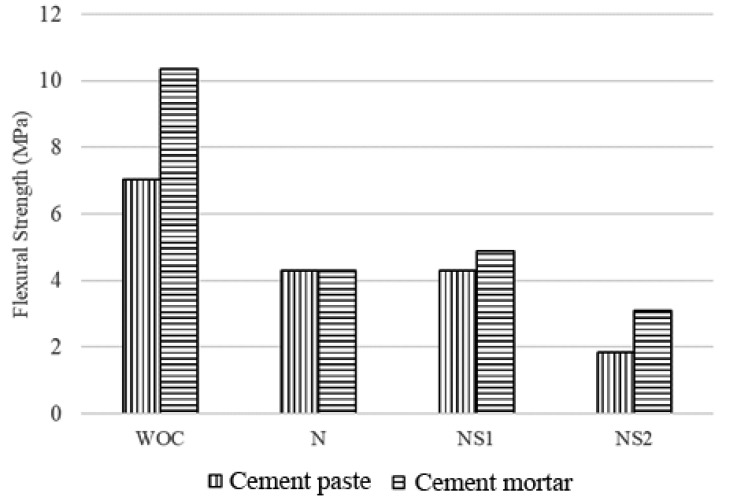
Flexural strength comparison between cement paste and mortars at crack initiation (after 7 days of curing, before self-healing). Samples without any capsules are designated as WOC.

The maximum average load applied on the mortars increased from 977 N to 1439 N with the incorporation of sand into the base matrix. As a result, the flexural strength of the cement mortars increased by 32.12% in comparison to the cement paste (Figure 11). This can be attributed to slower crack propagation in cement mortars due to the presence of sand [68].

The presence of CaAlg capsules deteriorated the flexural strength of both cement paste and mortars. The incorporation of N capsules into cement paste and mortars decreased their flexural strength by 38.96% and 58.50%, respectively. The reduction in flexural strength can be primarily attributed to the water uptake capacity of the CaAlg capsules. Previous research shows that CaAlg can swell up to three times its initial diameter when put into a cement slurry solution, and that this ability is beneficial for spore germination and the crack bridging [41]. However, in a realistic environment, the water retained in the capsules would be systematically released into the matrix during dry periods and hydrate the cement matrix, thus facilitating internal curing and improving the mechanical strength of the structure. However, drying, shrinking CaAlg capsules would leave voids in the base matrix that can compromise the structural integrity of cement and fasten the propagation of micro-cracks [69]. The initial loss of flexural strength with the addition of CaAlg capsules is certainly a disadvantage that should be examined and improved in future research. Such research can examine the impact of CaAlg capsules on reinforced cementitious materials to establish an acceptable quantity of CaAlg as a concrete additive based on the loss of initial strength and subsequent regain after self-healing. The loss of flexural strength observed in this study due to the presence of CaAlg capsules closely adheres to previous findings [44].

The NS1 capsules did not have a large impact on the flexural strength of the cement paste in comparison to the N capsules (Figure 11). The flexural strength results depicted an increase in strength from 4.3 to 4.9 MPa with N and NS1 capsules in the cement mortars, respectively. Therefore, the incorporation of 10^5^ spores/mL in the CaAlg capsules caused the flexural strength of cement mortars to increase by 11.81% compared to the N capsules. This increase could be a result of CaCO_3_ precipitates due to MICP as early as 7 days after immersion in water. The sand present in the cement mortars may provide better nucleation sites for crystal growth resulting in a higher flexural strength in comparison to the cement paste under similar conditions. The lack of a meaningful flexural strength difference between N and NS1 paste and mortars could also suggest that CaAlg capsules retain the self-healing agents after mortar preparation, since the release of such materials into concrete would retard the hydration and the physical properties of the material. This hypothesis is supported by the excellent spore retention ability of CaAlg capsules.

The capsules were incorporated at a concentration of 5% by volume of the mortar. This translates to the incorporation of an average of 4.5% of capsules by the mass of cement in the specimens, which resulted in an average decrease in flexural strength of 38.96% and 58.50% for N-cement paste and N-cement mortars, respectively (Figure 11). These results adhere to previous findings where a 15% reduction in compressive strength was obtained for the incorporation of 1% CaAlg capsules by mass of the cement [44].

The flexural strength of both cement paste and mortars reduced when the spore concentration in the capsules was increased to 10^7^ spores/mL (Figure 11). The NS2 capsules corresponded to a 73.84% and 70.10% reduction in flexural strength for cement paste and mortars, respectively. Similar results have been observed from previous research where the direct incorporation of *B. sphaericus* cells into mortar specimens at concentrations greater than 10^7^ cells/mL resulted in the systematic decrease in the compressive strength, which was attributed to the increase in water uptake due to increasing cell concentration in the mortar [70]. This will lead to an increase in the water uptake ability of the porous CaAlg capsules, leading to a larger decrease in the flexural strength.

#### 3.6.2. Flexural Strength of Cement Paste and Mortars after Self-Healing

**Figure 12:**
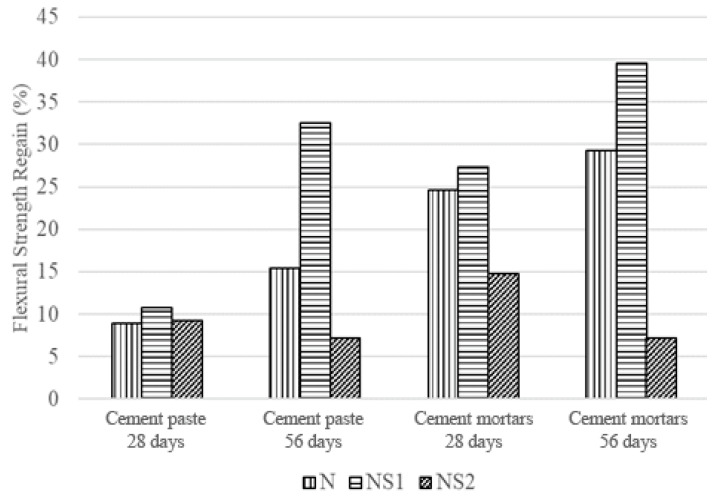
Flexural strength regains in cement paste and mortars after 28 and 56 days of incubation.

Figure 12 displays the flexural strength of the cement paste and mortars after 28 and 56 days of wet–dry cycle incubation. Flexural strength regain was determined by comparing the flexural strength of the specimen before and after the self-healing period. The self-healing period commenced after crack initiation and start of the wet–dry cycle incubation. After 28 days of wet–dry incubation, both the NS1-cement paste and NS1-cement mortars showed higher strength regain than N-cement paste and N-cement mortars, with NS1-cement and NS1-cement mortars recovering 10.8% and 27.3% of their original flexural strength, while N-cement paste and N-cement mortars recovered 8.9% and 24.6% of their flexural strength. The difference in strength regains became less prominent when comparing cement paste and mortars after 56 days of incubation, with 32.5% and 39.6% of flexural strength regain recorded for NS1-cement paste and NS1-cement mortars, respectively (Figure 12). N-cement paste and N-cement mortars recovered 15.4% and 29.3% of their flexural strength after 56 days.

The flexural strength regains results show that most of the self-healing for NS1-cement paste occurred in the latter 28 days of incubation, accounting for 67% of the recovery. Meanwhile, most of the self-healing that occurred in the NS1-cement mortars took place in the first 28 days of incubation, accounting for 69% of the recovery. The 58% recovery recorded in N-cement paste happened in the first 28 days, while 84% of the strength recovery observed in N-cement mortars occurred in the latter 28 days of wet–dry incubation.

The accelerated and improved flexural strength recovery of the NS1-cement mortars compared to N-cement mortars is undoubtedly due to bacteria-driven CaCO_3_ precipitation, which heals the crack that was introduced at day 0 of incubation, blocks the pores and fills the voids of the cement matrix, and heals micro-cracks that form in the structure, preventing their propagation. NS1-CaAlg capsules serve as CO_3_^2−^ generators embedded inside the cement matrix, resulting in faster and higher concrete self-healing.

The strength recovery difference between N-cement paste and N-cement mortars (13.9%) is almost double the strength recovery difference between the bacterial specimens (7.1%) after 56 days of incubation.

The difference in strength recovery between cement paste and mortars can be attributed to the presence of sand when non-bacterial (N-cement paste and N-cement mortar) and bacterial mortars (NS1-cement paste and NS1-cement mortar) are compared separately. Sand particles can provide additional crystallization surfaces for the CO_3_^2−^ ions diffusing out of the NS1-CaAlg capsules to react with the Ca^2+^ ions native to the cement environment, which could explain the higher strength recovery observed in NS1-cement mortars than NS1-cement paste. 

The NS2 capsules incorporated into cement paste and mortars did not result in any strength recovery. Although a slight increase in flexural strength could be observed after 28 days of being subjected to wet and dry cycles, the flexural strength of the specimens appeared to reduce after 56 days (Figure 12). Since the NS2 capsules have an increased spore concentration, it was considered that a higher nutrient concentration was required for self-healing. This hypothesis was tested by assessing the self-healing potential of NS2 capsules with increased nutrient concentrations, but no change was observed in the self-healing function of the modified NS2 capsules (data not shown). Therefore, it can be concluded that the spore concentration of 1.97 × 10^7^ is not suitable for the CaAlg capsules utilized in this research.

Our results show that the CaAlg capsules utilized in this research can protect and retain the bacteria at least up to 56 days under fluctuating conditions, while the incubation media (water) was changed daily, and the water-soluble nutrients were maintained. This point is certainly interesting, since the retention of water-soluble nutrients such as the peptide-based nutrient broth used in this research inside the CaAlg capsules remains speculative and untested, but it is positively reinforced by our observations. Future studies should focus on the retention of water-soluble nutrients in CaAlg capsules and how this property can be modified. The results suggest faster and more effective MICP-based self-healing in cement mortars. 

The addition of the N capsules resulted in a net flexural strength loss of 23.6% and 29.2% for the cement paste and mortars after 56 days, respectively. Meanwhile, the addition of the NS1 capsules resulted in a net flexural strength loss of 5.5% and 13.4% for the cement paste and mortars after 56 days, respectively. Overall, the initial loss of flexural strength due to CaAlg capsule addition is too high to be compensated by microbial CaCO_3_ precipitation, and further research is required to address this issue. Since the self-healing potential of this system is promising, perhaps future modifications can be done on the material properties of CaAlg by optimizing factors such as swelling and capsule size, which are known to impact the strength loss in cementitious materials [44]. While the lower net flexural strength loss of NS1-cement paste and NS1-cement mortar specimens is likely due to the higher CaCO_3_ precipitation rate in the specimens, examining the potential of reinforcing cement additives to counteract the strength loss by CaAlg addition may be an interesting undertaking [12].

#### 3.6.3. Visualization of Crack Healing

**Figure 13:**
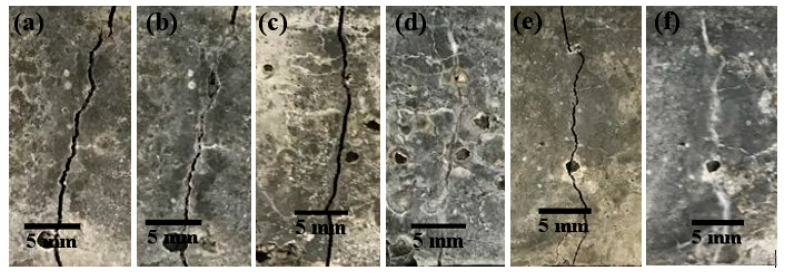
Examples of partially healed cracks after 28 days of wet and dry cycles; (**a**,**c**) before healing with N and NS2, respectively; (**b**,**d**) after healing with N and NS2, respectively; Example of complete healing of cracks after 28 days of wet and dry cycles; (**e**) before healing (**f**) after healing with NS1 capsules.

**Figure 14:**
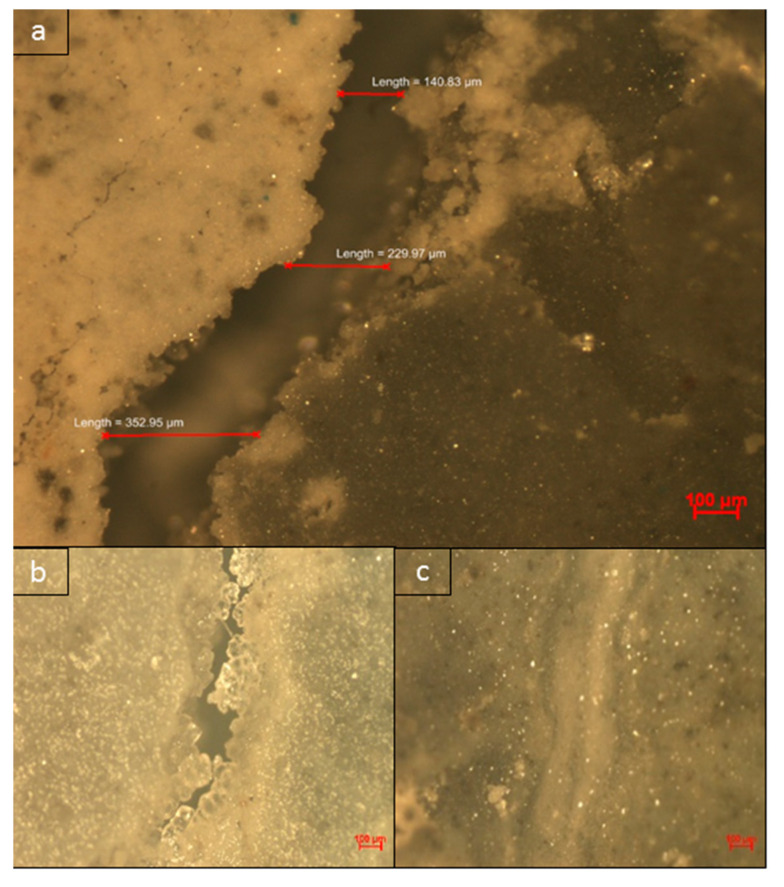
5× magnification images of cracks; (**a**) initial crack after 7 days of curing; (**b**) partially healed section of a crack; (**c**) completely healed section of a crack in NS1-cement mortar after 28 days.

Crack-healing visualization was done by imaging the surface of the cracks before and after the self-healing. Since the visualization of crack closures for all the specimen were similar, examples of partially and completely healed cracks have been provided in Figure 13 and Figure 14. The images captured after 28 days of wet and dry cycle incubation show completely healed cracks when the NS1 capsules were used. However, a few points along the crack surface were left unhealed in specimens containing N capsules. Therefore, the presence of bacteria can be seen to provide more efficient crack closure. Although NS2 capsules contained an increased spore concentration, the crack surfaces depicted unhealed spaces similar to the cracks on mortars containing N capsules. Therefore, either the increased spore concentration could not be sustained once they germinated to form a vegetative cell with a nutrient broth concentration of 8 g/L, or the increased spore concentration required water or oxygen for germination and growth, which was not available due to the dry cycles or the rate of their diffusion into the CaAlg capsules, hindering their growth.

After 56 days of being subjected to wet and dry cycles, all the crack at the surfaces in both cement paste and mortars containing N, NS1, and NS2 capsules were completely filled. This suggests that the sustained internal curing offered by CaAlg capsules regardless of the presence of bacteria or sand is enough for crack sealing, and based on the crack observations at 28 days, it is visible that bacterial activity can noticeably accelerate this process. Even though the cracks appear to be completely sealed at the surface, we could not comment on the healing of the crack depth. Full crack sealing would restrict airflow into the crack depths to the current passing through the interconnected pores of the cement matrix, which could slow bacterial activity and MICP. On the other hand, *B. pseudofirmus* is a facultative anaerobe, which means that it does not require a constant, ample supply of O_2_ to function. The full scope of MICP-based concrete self-healing by anaerobic and facultative anaerobic bacteria such as *B. pseudofirmus* should receive further research attention, as it could help address challenges such as the healing of deep cracks and increased efficiency and prolonged self-healing service of the concrete self-healing systems.

Magnified images of the healed cracks were obtained using light microscopy imaging and showed that the completely healed cracks appeared to be cemented together (Figure 13 and Figure 14c). White crystalline structures could be observed on the edges of partially healed cracks, which indicates the ongoing process of CaCO_3_ precipitation in the concrete self-healing process (Figure 13 and Figure 14b).

### 3.7. Morphological Analysis of Cracks after Self-Healing

The CO_2_ produced by the metabolic activity of the bacterial cells is transformed into CO_3_^2−^ ions in the high alkaline environment present in the concrete. Then, the CO_3_^2−^ ions react with Ca^2+^ ions to form CaCO_3_. After the initial precipitation of CaCO_3_, a concentration gradient is created between the capsule and the surrounding concrete matrix due to a reduction in CO_3_^2−^ ions. Therefore, the newly formed CO_3_^2−^ ions migrate to the surface of the capsule, resulting in a greater number of precipitates at the capsule surface [41]. Therefore, to effectively observe the morphology of the CaCO_3_ precipitates, FE-SEM images were mostly taken from the surface of the capsules. Images were obtained from the surface of N and NS1 capsules incorporated in cement paste and mortars.

There are three different forms of anhydrous polymorphs CaCO_3_ that can be found in nature. In the order of thermodynamic stability, they are vaterite, aragonite, and calcite [52]. The different polymorphs can be easily identified by their distinctive shapes. Vaterite has a unit cell of hexagonal shape. These precipitates can be found as hexagons, polycrystalline spherical shapes, and thin plates arranged in flower-like clusters [71,72,73]. Aragonite can be mostly observed in the form of thin needle-like precipitates with an orthorhombic single-crystal structure. Calcite, which is the most stable form of precipitates, can be found largely in cubic-shaped precipitates owing to its rhombohedral unit cell structure [16,71].

**Figure 15:**
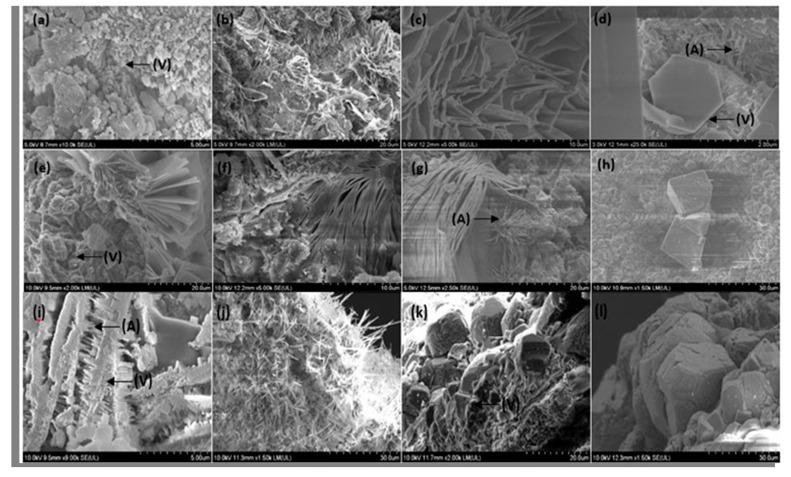
Precipitates observed on the surface of CaAlg capsules after 28 days of self-healing; (**a**–**d**) N capsules in cement paste; (**e**–**h**) NS1 capsules in cement paste; (**i**) N capsules in cement mortar; (**j**–**l**) NS1 capsules in cement mortar; arrows labeled A, V, C refer to aragonite, vaterite, and calcite precipitates, respectively.

Vaterite precipitates were found on the surfaces of N and NS1 capsules in both cement paste and mortars. Spherical vaterite of 0.5 μm was observed on N capsules in cement paste and mortars (Figure 15a,i). Similarly, spherical precipitates of 4 μm were observed on the surfaces of spore containing capsules in cement paste and mortars (Figure 15i,k). Hexagonal precipitates were observed on the surfaces of N capsules in cement paste and had an approximate width of 1.6 μm (Figure 15c). Plate-like vaterite precipitates arranged in flower-like clusters had widths of 0.1–0.5 μm and 8–10 μm on N and NS1 capsules in cement paste, respectively (Figure 15b,c,e). The stacked plate-like precipitates observed on the NS1 capsules in cement paste with a width of 1 μm and lengths ranging from 10 to 30 μm (Figure 15f,g) are also consistent with larger plate-like vaterite precipitates arranged in flower-like clusters but packed more closely together.

The aragonite precipitates on the surface of N capsules had lengths of 0.8 μm and 2 μm when incorporated in cement paste (Figure 15d) and cement mortars (Figure 15i), respectively. The surfaces of aragonite precipitates on NS1 capsules had lengths of 4 μm and 9 μm in cement paste and mortars, respectively (Figure 15g,j).

Calcite precipitates were only observed on the surface of N capsules when incorporated in cement mortars and had an approximate length and width of 0.5 μm (Figure 15i). The surfaces of NS1 capsules contained calcite precipitates when incorporated in both cement paste and mortars. The calcite precipitates in cement paste had an approximate length and width of 18 μm (Figure 15h). The NS1 capsules in cement mortars had calcite precipitates that could be dimensioned in the range of 16–30 μm (Figure 15k,l). SEM analysis conducted in previous research has also confirmed the presence of rhombohedral calcite precipitates resulting from MICP [37,52].

From the SEM results, it can be concluded that the process of MICP not only facilitates larger and a more abundant supply of CaCO_3_ precipitates but also more stable forms of precipitates. Previous research has discovered that in comparison to aragonite, calcite contains a higher bonding strength with the hydrates of cement [74]. This would correspond to the improved mechanical properties and durability of cement paste and mortars. Therefore, the SEM results corroborate the flexural strength results where improved flexural strength is observed due to MICP after self-healing. The more abundant and more stable forms of precipitates observed on the surfaces of N and NS1 capsules when in cement mortars as opposed to cement paste further confirm that the presence of sand awards better nucleation sites for CaCO_3_ crystal growth.

## 4. Conclusions

We effectively encapsulated *B. pseudofirmus* spores in CaAlg capsules, assessing the success of encapsulation and spore retention in the capsules, as well as the precipitation potential of the immobilized bacteria. The significant majority of the spores survived the encapsulation process, and nearly all were retained in capsules under simulated cement mixing conditions. While CaAlg capsules prepared with either CaL or CaCl_2_ displayed signs of degradation after exposure to CF, this was not observed in capsules placed inside cement samples. Hence, CaAlg capsules are suitable carriers for *B. pseudofirmus* spores. The encapsulated bacteria successfully precipitate CaCO_3_ when exposed to environmental elements in the presence of Ca^2+^ under alkaline conditions. CaAlg capsules do not require physical damage to initiate the healing process. Healing can initiate when water and oxygen seep through the cracks. Although CaAlg capsules initially reduced the flexural strength of the cement paste and mortars, the capsules successfully healed small cracks in the cement paste and mortars after 56 days of exposure to wet–dry conditions, resulting in a flexural strength recovery of 39.6% in cement mortars and 32.5% in cement paste. CaAlg capsules are a promising carrier material for concrete self-healing, as they provide effective encapsulation to both bacteria and nutrients up to 56 days. Future research could be directed to the survival estimation methods to assess the survival of spores after incorporation into cementitious materials, to quantitatively assess the nutrient retention ability of the capsules, and to optimize the quantity and size of capsules to achieve desirable self-healing without significant physical strength reduction, as the self-healing behavior observed in this study was not enough to counteract the initial cement strength loss due to the addition of CaAlg. Furthermore, the functionality of CaAlg capsules for other purposes such as marine applications is another promising area for future research as well.

## Figures and Tables

**Table 1:** Composition and description of prepared CaAlg capsules.

Capsule	Capsule Description	Nutrient Broth (8 g/L)	Bacterial Spore (Cells/mL Na–Alginate Solution)
O	Capsules with no additives	-	-
S	Capsules containing only spores	-	8.40 × 10^5^
N	Capsules containing only nutrients	√	-
NS1	Capsules containing nutrients and spores	√	8.40 × 10^5^
NS2	Capsules containing nutrient and increased spores	√	7.88 × 10^8^

**Table 2:** Summary of the cement paste and mortars.

Sample Type	Capsule Type	Sections without Capsules			Section with Capsules			
		Cement (wt %)	Water (wt %)	Sand (wt %)	Cement (wt %)	Water (wt %)	Sand (wt %)	Capsule (wt %)
WOC *-cement paste	-	74.08	25.92	-	-	-	-	-
N-cement paste	N	74.07	25.93	-	72.37	25.28	0.00	2.34
NS1-cement paste	NS1	74.07	25.93	-	72.37	25.28	0.00	2.34
NS2-cement paste	NS2	74.07	25.93	-	72.37	25.28	0.00	2.34
WOC-cement mortar	-	42.55	14.89	42.55	-	-	-	-
N-cement mortar	N	42.56	14.89	42.56	41.47	14.53	41.47	2.53
NS1-cement mortar	NS1	42.56	14.89	42.56	41.47	14.53	41.47	2.53
NS2-cement mortar	NS2	42.56	14.89	42.56	41.47	14.53	41.47	2.53

* WOC: without capsules.

**Table 3:** The average survival of encapsulated spores from de-cross-linked S, NS1, and NS2 capsules.

Capsule Type	Average Spore Concentration (Cells/mL)	Expected Values (Cells/mL)
S	8.47 × 10^4^ ± 1.84 × 10^4^	7.39 × 10^4^
NS1	7.62 × 10^4^ ± 2.21 × 10^4^	7.39 × 10^4^
NS2	1.97 × 10^7^ ± 5.65 × 10^6^	6.93 × 10^7^

## Supplementary Materials

The following are available online at https://www.mdpi.com/1996-1944/13/17/3711/s1, S1: Calculation of theoretical capsule viability for NS1 capsules, Figure S1: Results of statistical analysis: one-way ANOVA on viability of S and NS1 capsules and the theoretical capsule viability calculated in S1. Significance in difference indicates significantly different spore viability (*p* < 0.05), Figure S2: Optical microscopy images of capsules placed inside cement samples and cured for 14 days in DW. (a) NS1 capsule after 14 days inside cement ×40 magnification (b) NS1 capsule after 14 days inside cement ×100 magnification (c) N capsule after 14 days inside cement ×40 magnification (d) N capsule after 14 days inside cement ×100 magnification (e) O capsule after 14 days inside cement ×40 magnification (f) O capsule after 14 days inside cement ×100 magnification.
