# Supplementary material for: Biological Self-Healing of Cement Paste and Mortar by Non-Ureolytic Bacteria Encapsulated in Alginate Hydrogel Capsules
Source: Materials (Basel). 2020 Aug 22;13(17):3711. doi: 10.3390/ma13173711 (PMC7504608; doi:10.3390/ma13173711)
Supplement: Supplementary file 1 [file materials-13-03711-s001.pdf]

materials

MDPI

Supplementary information

Biological Self-Healing of Cement Paste and Mortar by Non-Ureolytic Bacteria Encapsulated in Alginate Hydrogel Capsules

Mohammad Fahimizadeh [1], Ayesha Diane Abeyratne [1], Lee Sui Mae [2], R. K. Raman Singh [3] and Pooria Pasbakhsh [1,*]

[1] School of Engineering, Monash University Malaysia, Bandar Sunway 47500, Malaysia; Mohammad.Fahimizadeh@monash.edu (M.F.); ayeshaabeyratne@gmail.com (A.D.A.)
[2] School of Science, Monash University Malaysia, Bandar Sunway 47500, Malaysia; lee.sui.mae@monash.edu
[3] Department of Chemical Engineering, Department of Mechanical and Aerospace Engineering, Monash University, Clayton 3168, Australia; raman.singh@monash.edu
* Correspondence: pooria.pasbakhsh@monash.edu

Received: 24 June 2020; Accepted: 20 July 2020; Published: date

S1. Calculation of theoretical capsule viability for NS1 capsules

Average viability of 3 spore suspensions used for preparation of biological replicate capsules was determined to be 3.36 × 10^7 cells/mL for S and NS1 capsules, and 3.16 × 10^{10} cells/mL for NS2 capsules. 0.25 mL of spore suspension was added to 9.75 mL of sodium alginate solution.

Viability of the encapsulation solution: $C_1V_1=C_2V_2$

S and NS1: $(3.36 \times 10^7) \times 0.25 = C_2 \times 10$; $C_2 = (3.36 \times 10^7) \times 0.025 = 8.40 \times 10^5$ cells/mL

NS2: $(3.16 \times 10^{10}) \times 0.25 = C_2 \times 10$; $C_2 = (7.88 \times 10^8) \times 0.025 = 7.88 \times 10^8$ cells/mL

Average number of capsules obtained from 10 mL of encapsulation solution was 114 capsules. Hence, each capsule is made from 10/114 = 0.088 mL of encapsulation solution.

5 capsules were dissolved in 5 mL of citrate-EDTA buffer. Hence, the theoretical viability of spores in a single capsule is: $C_1V_1=C_2V_2$

S and NS1: $(8.40 \times 10^5) \times (5 \times 0.088) = C_2 \times 5$; $C_2 = (8.40 \times 10^5) \times 0.088 = 7.39 \times 10^4$ cells/mL

NS2: $(7.88 \times 10^8) \times (5 \times 0.088) = C_2 \times 5$; $C_2 = (1.97 \times 10^7) \times 0.088 = 6.93 \times 10^7$ cells/mL

Standard deviation of this theoretical value was calculated based on the standard deviation of the viability of the 3 spore suspensions, which was 2.44 × 10^7 cells/mL

$(2.44 \times 10^7) \times 0.025 \times 0.088 = 5.3 \times 10^4$

Univariate Analysis of Variance

Between-Subjects Factors

		Value Label	N
Type	1.00	NS1	3
	2.00	S	3
	3.00	Theoretical	3

Tests of Between-Subjects Effects

Dependent Variable: Viability

Source	Type III Sum of Squares	df	Mean Square	F	Sig.
Corrected Model	190058756[a]	2	95029377.78	.051	.950
Intercept	5.517E+10	1	5.517E+10	29.813	.002
Type	190058755.6	2	95029377.78	.051	.950
Error	1.110E+10	6	1850708267		
Total	6.647E+10	9			
Corrected Total	1.129E+10	8			

a. R Squared = .017 (Adjusted R Squared = -.311)

Post Hoc Tests

Type

Multiple Comparisons

Dependent Variable: Viability
Tukey HSD

(I) Type	(J) Type	Mean Difference (I-J)	Std. Error	Sig.	95% Confidence Interval	
					Lower Bound	Upper Bound
NS1	S	-8433.3333	35125.56777	.969	-116208.2210	99341.5544
	Theoretical	2240.0000	35125.56777	.998	-105534.8877	110014.8877
S	NS1	8433.3333	35125.56777	.969	-99341.5544	116208.2210
	Theoretical	10673.3333	35125.56777	.951	-97101.5544	118448.2210
Theoretical	NS1	-2240.0000	35125.56777	.998	-110014.8877	105534.8877
	S	-10673.3333	35125.56777	.951	-118448.2210	97101.5544

Based on observed means.
The error term is Mean Square(Error) = 1850708266.667.

Figure S1. Results of statistical analysis: one-way ANOVA on viability of S and NS1 capsules and the theoretical capsule viability calculated in S1. Significance in difference indicates significantly different spore viability ($P < 0.05$).

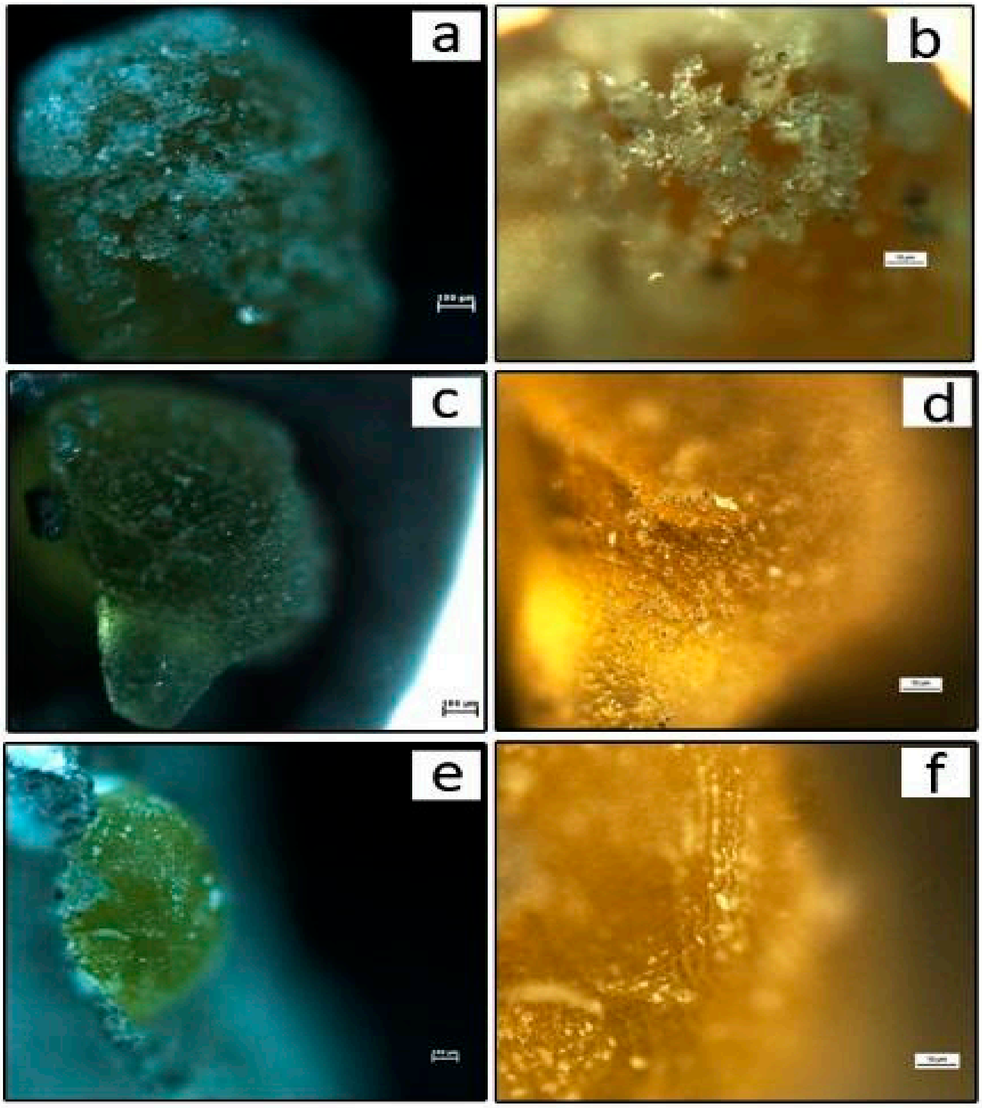

Figure S2. Optical microscopy images of capsules placed inside cement samples and cured for 14 days in DW. (**a**) NS1 capsule after 14 days inside cement ×40 magnification (**b**) NS1 capsule after 14 days inside cement ×100 magnification (**c**) N capsule after 14 days inside cement ×40 magnification (**d**) N capsule after 14 days inside cement ×100 magnification (**e**) O capsule after 14 days inside cement ×40 magnification (**f**) O capsule after 14 days inside cement ×100 magnification.

© 2020 by the authors. Licensee MDPI, Basel, Switzerland. This article is an open access article distributed under the terms and conditions of the Creative Commons Attribution (CC BY) license (http://creativecommons.org/licenses/by/4.0/).
